# Understanding Interdependencies between Mechanical Velocity and Electrical Voltage in Electromagnetic Micromixers

**DOI:** 10.3390/mi11070636

**Published:** 2020-06-29

**Authors:** Noori Kim, Wei Xuan Chan, Sum Huan Ng, Yong-Jin Yoon, Jont B. Allen

**Affiliations:** 1 Department of Electrical and Electronic Engineering, Newcastle University in Singapore, 172A Ang Mo Kio Avenue 8, ♯05-01 SIT@NYP Building, Singapore 567739, Singapore; 2 Department of Biomedical Engineering, National University of Singapore, 21 Lower Kent Ridge Rd, Singapore 119077, Singapore; biecwx@nus.edu.sg; 3 Singapore Institute of Manufacturing Technology, 2 Fusionopolis Way, Singapore 138634, Singapore; shng@simtech.a-star.edu.sg; 4 Department of Mechanical Engineering, Korea Advanced Institute of Science and Technology, Daejeon 34141, Korea; yongjiny@kaist.ac.kr; 5 Department of Electrical and Computer Engineering, University of Illinois at Urbana-Champaign, Urbana, IL 61801, USA; jontalle@illinois.edu

**Keywords:** micromixers, acoustic micromixers, active micromixers, electromagnetic micromixers, voice-coil mixers, mixers, anti-reciprocity, electrical impedance, mechanical velocity, gyrator, electro-mechanical systems

## Abstract

Micromixers are critical components in the lab-on-a-chip or micro total analysis systems technology found in micro-electro-mechanical systems. In general, the mixing performance of the micromixers is determined by characterising the mixing time of a system, for example the time or number of circulations and vibrations guided by tracers (i.e., fluorescent dyes). Our previous study showed that the mixing performance could be detected solely from the electrical measurement. In this paper, we employ electromagnetic micromixers to investigate the correlation between electrical and mechanical behaviours in the mixer system. This work contemplates the “anti-reciprocity” concept by providing a theoretical insight into the measurement of the mixer system; the work explains the data interdependence between the electrical point impedance (voltage per unit current) and the mechanical velocity. This study puts the electromagnetic micromixer theory on a firm theoretical and empirical basis.

## 1. Introduction

Fluid mixing techniques are ubiquitous in microfluidic applications. The spectrum of use has been broadened in many areas, such as biological, medical, and chemical research and industries [1,2]. Micromixers are typical devices for mixing a micro amount of fluids with various mixing principals. There are two types of micromixers, active and passive. The passive micromixers do not need power sources; they use pressure to guide fluid blending. However, active micromixers require actuators, which use an external source of energy in the form of acoustic, electrokinetic, electrowetting, magnetic, electromagnetic, etc. [3].

Electromagnetic mixers utilise the Lorentz force; alternating voltages are applied to positive and negative electrical terminals of the devices to induce the alternating current. The fluctuating electrical motions cause complicated fluid motions, which generate multi-micro streams and enhance the mixing efficiency [4]. An electromagnet was used to generate transient interactive flows to enhance micromixing by Chih-Yung Wen and Lung-Ming [5]; the authors observed interesting miscible maze-like patterns. They presented a ferrohydrodynamic micromixer that utilises low-voltage and low-frequency properties. Numerical studies on electromagnetic mixers were also performed to characterise magneto-hydrodynamic micro-mixers [4,6,7]. Yiping Chen and Kim [7] introduced an electromagnetic fluidic device to enable both mixing and pumping functions by the Lorentz force, which was induced by the current and applied magnetic field. Pengwang et al. [8] reviewed and compared many types of actuators to be used in the micro-electromechanical system (MEMS) area, including electromagnetic actuators. Although the main objective of the article focuses on MEMS-based scanning micromirrors, general actuator principles apply the same in the MEMS area. Compared to other types of actuators, the electromagnetic actuator requires lower driving voltage, but can achieve a more significant driving force. However, they may need external magnets, which can increase the system size and create electromagnetic interference.

The acoustic mixing techniques provide highly efficient and controlled mixing results with little restrictions on the types of fluids used. The acoustic micromixers blend fluids using devices that induce micro-streams. They use various mediums, such as trapped micro air bubbles [9], a thin solid plate [10], or a piezoelectric membrane [11,12,13,14,15] to transfer the acoustic energy. Chan et al. [16] demonstrated controlling acoustic energy propagation in a microfluidic chip via frequency selectivity. They employed a voice coil as an electromagnetic force-driven acoustic actuator. The electromagnetic and acoustic domains were coupled by the “moving-coil” component.

Kim et al. [17] presented a microfluidic mixer, which consisted of a chamber and an acoustic actuator. The voice coil actuator was electromagnetically and mechanically coupled to the cylindrical chamber. It converted a periodic input electrical signal at an optimum operating frequency to vibratory mechanical stress entering the chamber. They also demonstrated that an optimum operating frequency of the input electrical signal could be determined by sweeping frequencies, measuring the corresponding impedance in the frequency range, and selecting the sweet-spot operating frequency based on the impedance-frequency plot.

Commonly, the mixing efficiency of micromixers is analysed by mixing parameters such as time, length, and the mixing index [18,19], and these parameters depend on the ability of the transducer system (which includes the energy source, transducers, and membrane/transmission materials). To optimise transducer efficiency, there are generally a few approaches. As a theoretical approach, system modelling is performed for numerical simulations using various software such as ANSYS/CFX, FLUENT, and COMSOL Multiphysics or written codes, for instance the lattice Boltzmann technique [18]. The computing simulation is critical to estimating and optimizing all mixing parameters, especially for the experiments’ initial conditions. As a direct practical approach, a laser Doppler vibrometer or a micro-force sensor is employed. However, these approaches require additional costs such as computational resources or system installation. With an indirect approach, the efficiency of the transducer is combined with the other parameters influencing the mixing efficiency, and measurement using a tracer (such as a colour dye) combined with an image processing technique is used to estimate the transducer efficiency [17]. Although mixing parameters based on the experiment would predict a sufficiently accurate result, dead zones may exist; the image-based tracer analysis can be a suitable method for ascertaining the degree of mixing. External image monitoring devices can be considered as add-ons to support mixing efficiency analysis; however, it adds costs to the system installation [16,18]. Moreover, more trial and error are required to optimise both the transducer and the mixing system together.

One unique approach introduced by Kim et al. [17] was to use input impedance measurement from electrical terminals of the systems to measure the velocity response of the membrane. They demonstrated the mixing degree detection from the electrical impedance measurement. From a practical point of view, the experimental installation was simple and affordable compared to the conventional methods (of tracking the mixing degree) discussed above. In this paper, we elucidate the physical foundation underlying the micromixer’s electromagnetic coupling by adopting the principle of a component called a gyrator. Starting by introducing two electrical coupling devices, a transformer and a gyrator, we derive a correlation between the electrical driving point impedance and mechanical velocity in a two port electromagnetic system, the acoustic voice-coil micromixer. We also discuss experimentally measured results to support the theoretical concept.

## 2. Theories and Methods

A systematic way to understand different fields in engineering or physics begins by generalising the areas. Fields (or domains or modalities, i.e., electric, acoustic, mechanic, etc.) are analogous to each other. Two conjugate variables exist in pairs in each field, a generalised force and a flow. The two variables are used to characterise a modality by their product and their ratio. They could be either a vector (in bold) or a scalar and also can vary spatially. A product of these two variables defines the power, while a ratio establishes the impedance, which is usually determined in the frequency domain. Some examples of the conjugate variables in each modality are described in Table 1.

One can define a system using a single modality (one port system) or a combination of them. Typical examples of the combined modality systems are electroacoustics and electromechanics, speakers, and motors, respectively. An electromagnetic system is under a subcategory of the electro-mechanical systems. It couples electrical and mechanical domains through the electromagnetic field (Table 1).

To combine the modalities, the port network concept and coupling components are required. Regardless of the modalities, there is a law that applies to the every system, the law of the conservation of energy [20,21]:

(1) $$e\left(t\right)\equiv \int_{-\infty }^{t}power\left(t\right)dt\geq 0,$$

where the total delivered energy e(t), which is an integration of the power over time, should be greater than (or equal to) zero, and power(t) is work done per unit time defined as a potential multiplied by a net flow. In other words, Equation 1 means we cannot have more energy than we supply.

A transformer and a gyrator are two standard modality-coupling units. Both of them are defined as electrical components having transmission (ABCD) matrices; cascading ABCD matrices of circuit components are a popular tool to analyse circuits [22,23,24,25]. The transformer is a typical element that links one modality to another in a one-to-one manner; a flow in one modality is linear to the flow in the other field. Ideal and non-ideal cases of the transformer’s theory and the ABCD matrices are established well in classic works in the literature [23]. In the non-ideal case, mutual coupling factors between two adjacent circuits are considered [20,23,26].

McMillan [27] introduced a two port network that violates reciprocity; two years later, Tellegen [28] defined an ideal gyrator as the 5th electrical component to support the anti-reciprocal characteristics of the system. A unique thing about this gyrator is that it can model an electromagnetic (EM) network without using the mobility analogy, which is effective mathematically, but an anti-intuitive method to describe an EM system. The variables of each modality can be represented without modification as they have a gyrator as an EM coupler between the electrical and mechanical terminals.

**Figure 1:**
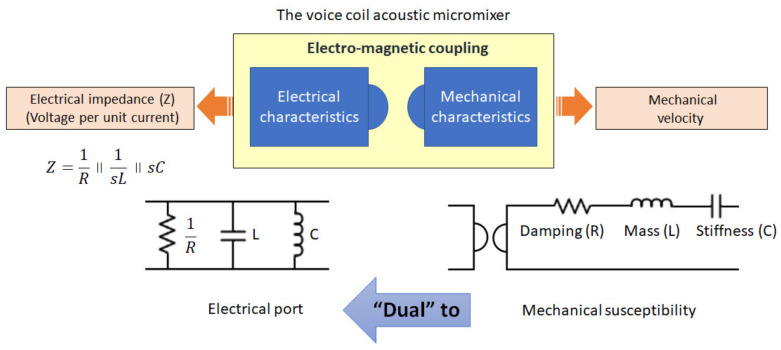
Mobility networking through electromagnetic coupling in the voice-coil acoustic micromixer. Without employing the gyrator for EM circuits, the mechanical components become dual when seen on the electrical side of the network.

The mobility (dual) analogy must be appreciated to model an EM system with a transformer. Figure 1 explains the dual method; the two circuit representations are equivalent to each other. If the gyrator is not used in the circuit, a series combination of the mass, damping, and stiffness in the mechanical side variables becomes a parallel relationship with the anti-reciprocation of each variable [29]. Furthermore, the flow and potential in one domain (i.e., U,F) become a potential and flow on the other side, respectively (i.e., Φ,I). Despite the usefulness of using a gyrator in modelling EM systems, this component has not become mainstream in EM circuit modelling and analysis.

Other than [25], only a few approaches have been made in EM system modelling with gyrators, mainly limited in power electronic fields. Yan and Lehman [30] demoted the benefit of using a gyrator in the EM modelling approach. In their work, a simplified modelling method was introduced for DC/DC converters using an extended capacitor-gyrator (C-G) modelling technique. They showed their modelling feasibility in complex core and winding structures. Young et al. [31] used the C-G to model a continuously variable series reactor (CVSR), which requires prudent planning to design. The authors also highlighted the convenience of using gyrators in their system modelling. Zhang et al. [32] demonstrated an improved C-G modelling method in power systems by taking the eddy current effect into account. They claimed that classical C-G modelling was not suitable; the eddy current impact must be considered in EM modelling. This point also has been demonstrated by Kim and Allen [25].

A two-port network, such as an electro-mechanical system, has Φ, *I*, *F*, and *U* as the system’s variables. A gyrator exists to couple the electric and mechanical sides. To specify this property, the impedance matrix of an ideal gyrator is employed:

(2) $$Z_{gyrator}=\left[\begin{array}{cc} 0 & -G\\ G & 0 \end{array}\right],$$

where $G=B_{0}l$ is the gyration coefficient, $B_{0}$ is the DC magnetic field, and *l* is the length of the wire. Thus:

(3) $$\left[\begin{array}{c} \Phi (\omega )\\ F(\omega ) \end{array}\right]=\left[\begin{array}{cc} 0 & -B_{0}l\\ B_{0}l & 0 \end{array}\right]\left[\begin{array}{c} I(\omega )\\ U(\omega ) \end{array}\right],$$

namely,

(4) $$\Phi \left(\omega \right)=-B_{0}lU\left(\omega \right)\;\mathrm{and}\;F\left(\omega \right)=B_{0}lI\left(\omega \right).$$

Taking the ratio of the two equations in Equation (4), an impedance is derived:

(5) $$\begin{array}{ccc} \frac{\Phi (\omega )}{B_{0}lI\left(\omega \right)} & = & \frac{-B_{0}lU\left(\omega \right)}{F(\omega )}\\ Z(\omega ) & = & \frac{\Phi (\omega )}{I(\omega )} \end{array}$$

When defining an impedance, the flow direction is defined as into the terminals; thus, *U* is defined as going into the network. Therefore, the minus sign of *U* in Equation (4) follows from Lenz’s law. Note that Equation (4) explains an ideal gyrator, considering only a DC magnetic field. Appreciating that the impedance is a concept in the frequency domain, an angular frequency symbol, ω, is omitted from this part and onward.

Considering only a single ideal gyrator element, we can obtain the velocity response via measuring the electrical impedance while performing a constant current sweep across frequencies,

(6) $$\begin{array}{ccc} Z_{idealG}\left(\omega \right) & = & \frac{-B_{0}lU\left(\omega \right)}{I} \end{array}$$

### 2.1. The Non-Ideal Gyrator

The non-ideal case of Equation (4) is considered from the basics of electromagnetism. Ulaby [33], Kim and Allen [34] described the induced emf (voltage ϕ(t)) as the sum of a transformer component ($\phi_{tr}\left(t\right)$) and a motional component ($\phi_{mot}\left(t\right)$), namely,

(7) $$\phi \left(t\right)=\phi_{tr}\left(t\right)+\phi_{mot}\left(t\right).$$

The transformer voltage is:

(8) $$\begin{array}{ccc} \phi_{tr}\left(t\right) & = & -(-\int \frac{\partial B(t)}{\partial t}\cdot \mathrm{dA})\\ & = & \frac{\partial \psi (t)}{\partial t} \end{array}$$

where ψ(t) is the magnetic flux. The voltage has an opposite direction from the emf, $\mathrm{emf}\equiv \oint E\cdot \mathrm{dl}=\int_{a}^{b}E^{\prime }\cdot \mathrm{dl}=-\phi \left(t\right)$. In the static case, the time-varying term is zero.

$\phi_{mot}$ represents the motion of the electrical voltage [33]. The voltage is associated with the motion from the other port (i.e., mechanical). In other words, the signal is observed from the mechanical side (motional voltage due to *u*). Note that this concept can be applied only in two port (or higher order) systems.

Derivation of $\phi_{mot}$ starts from the Lorentz magnetic force ($f_{m}$), acting on a moving charge *q* inside a magnetic field B with a velocity U,

(9) $$f_{m}=q\left(U\times B\right).$$

Then, the motion of the magnetic force from the electrical field $E_{mot}$ is $f_{m}=qE_{mot}$.The unit of *q* is in Coulombs (C) and $E_{mot}$ in (V/m) = (N/C) as 1 (V)≡1 (J/C) and 1 (N)=1 J/m. Therefore, qE stands for force with a unit of N. A positive charge (q>0, proton) is $1.602\times 10^{-19}$ C; thus, the charge of an electron (negative charge) is $-1.602\times 10^{-19}$ C. One coulomb of charge equals the charge that can light a 120-watt-bulb for one second. Therefore,

(10) $$\begin{array}{ccc} E_{mot} & = & \frac{f_{m}}{q}\\ & = & U\times B, \end{array}$$

where $E_{mot}$ is the motional electric field seen by the charged particle *q*, and its direction is perpendicular to both U and B.

Thus, the voltage $\Phi_{mot}$ is defined as the line integral of the corresponding electric field, which is $E_{mot}$ in this case,

(11) $$\begin{array}{ccc} \phi_{mot}\left(t\right) & = & -\oint_{C}E_{mot}\cdot \mathrm{dl}\\ & = & -\oint_{C}\left(U\times B\right)\cdot \mathrm{dl}. \end{array}$$

This term has been considered in the ideal gyrator.

Finally, the total voltage becomes:

(12) $$\begin{array}{ccc} \phi (t) & = & \phi_{tr}\left(t\right)+\phi_{mot}\left(t\right)\\ & = & \int \frac{\partial B(t)}{\partial t}\cdot \mathrm{dA}-\oint_{C}\left(U\times B\right)\cdot \mathrm{dl}. \end{array}$$

In the frequency domain, Equation (12) is rewritten as:

(13) $$\begin{array}{ccc} \Phi & = & s\Psi -BlU\\ & = & sL_{e}I-BlU, \end{array}$$

where s=jω, $L_{e}$ is a leakage inductance due to the leakage flux of a self-inductance in the electrical side, $\Psi =L_{e}I$.

Assuming a static DC magnetic field ($B_{0}$), then sΨ=0, and we find the ideal gyrator definition $\Phi =\Phi_{mot}=-UB_{0}l$ (Equation (4)). The frequency dependent term shown in Equation (13) (jωΨ and $j\omega L_{e}I$) is a non-quasistatic (dynamic) term that is not considered in an ideal gyrator. The minus sign for the other term −UBl is related to Lenz’s law.

Two types of magnetic fields exist in an electro-mechanical network: one is the DC magnetic field, and the other is the AC magnetic field. In the ideal gyrator formula, only the motional parts (or the DC magnetic field) of the variables (voltage and force) are considered, which dominate an EM system usually. The two modalities in the network (electrical and mechanical) share this DC magnetic field, which is shown in the motional part of each variable. For the non-ideal case, the transduction parts (or AC magnetic field) of variables along with the motional parts must be considered.

For a non-ideal gyrator, we rewrite the mixing system model (from Equation (5)) with the transformer voltage and mechanical impedance (membrane response), $Z_{m}$,

(14) $$\begin{array}{ccc} \Phi (\omega ) & = & -B_{0}lU\left(\omega \right)+j\omega L_{e}I\left(\omega \right) \end{array}$$

(15) $$\begin{array}{ccc} F(\omega ) & = & B_{0}lI\left(\omega \right)+Z_{m}U\left(\omega \right) \end{array}$$

(16) $$\begin{array}{ccc} Z(\omega ) & = & \frac{-B_{0}lU\left(\omega \right)}{I}+j\omega L_{e} \end{array}$$

When the transformer impedance is not small (i.e., $|\omega L_{e}I\left(\omega \right)|\approx |B_{0}l|$), we have to use a very small constant current, and the question of whether the velocity response in its reasonable operating condition can be reflected in the electrical impedance response might be raised. Therefore, we formulate the relationship between the velocity response and electrical impedance with another approach.

### 2.2. Velocity Frequency Response with Constant Voltage

From Equation (14),

(17) $$\begin{array}{ccc} \Phi (\omega ) & = & -B_{0}lU\left(\omega \right)+j\omega L_{e}\frac{\Phi (\omega )}{Z(\omega )}\\ U(\omega ) & = & \frac{\Phi (\omega )}{B_{0}l}\left[\frac{j\omega L_{e}}{Z(\omega )}-1\right] \end{array}$$

During a constant voltage frequency sweep (i.e., Φ(ω)=Φ),

(18) $$\begin{array}{ccc} U(\omega ) & = & \frac{\Phi }{B_{0}l}\left[\frac{j\omega L_{e}}{Z(\omega )}-1\right] \end{array}$$

As $\omega L_{e}$ is the same order as Z(ω), small changes in the electrical impedance Z(ω) will be reflected in the velocity response and vice versa.

To illustrate this concept in more detail, we substitute I(ω) via Equations (15) into (16) to obtain the impedance response,

(19) $$\begin{array}{ccc} Z(\omega ) & = & \frac{B_{0}lU\left(\omega \right)}{I(\omega )}+j\omega L_{e}\\ & = & \frac{-{\left(B_{0}l\right)}^{2}U\left(\omega \right)}{F\left(\omega \right)-Z_{m}U\left(\omega \right)}+i\omega L_{e} \end{array}$$

Without external force (i.e., no other mixing source in the vicinity or the clamped mechanical system that cannot move), the electrical impedance response in terms of the mechanical impedance is:

(20) $$\begin{array}{ccc} Z(\omega ) & = & \frac{{\left(B_{0}l\right)}^{2}}{Z_{m}}+i\omega L_{e} \end{array}$$

We also substitute Equations (20) into (17), to obtain the (constant voltage) velocity response in terms of the mechanical impedance,

(21) $$\begin{array}{ccc} U(\omega ) & = & \frac{\Phi }{B_{0}l}\left[\frac{j\omega L_{e}}{Z(\omega )}-1\right]\\ & = & \frac{\Phi }{B_{0}l}\left[\frac{j\omega L_{e}Z_{m}}{{\left(B_{0}l\right)}^{2}+i\omega L_{e}Z_{m}}-1\right]\\ & = & \frac{\Phi }{B_{0}l}\frac{-{\left(B_{0}l\right)}^{2}}{{\left(B_{0}l\right)}^{2}+i\omega L_{e}Z_{m}} \end{array}$$

When the mechanical impedance is much larger than the gyrator coefficient ($B_{0}l$),

(22) $$\begin{array}{ccc} Z(\omega ) & \approx & \frac{{\left(B_{0}l\right)}^{2}}{Z_{m}} \end{array}$$

and

(23) $$\begin{array}{ccc} U(\omega ) & \approx & \frac{-B_{0}l\Phi }{i\omega L_{e}Z_{m}} \end{array}$$

Then, Equation (22) can be rewritten as:

(24) $$\begin{array}{ccc} Z(\omega ) & \approx & \frac{-i\omega L_{e}B_{0}l}{\Phi }U\left(\omega \right). \end{array}$$

### 2.3. Mechanical Impedance

From the forced vibration equation, the force due to the thin membrane [35],

(25) $$\begin{array}{c} F_{m}=\omega \sum_{\phi }\zeta \left(\omega ,\phi ,\rho_{m},h,c,Y\right)\left(\omega_{r}^{2}-\omega^{2}\right)U \end{array}$$

where ζ is the membrane properties function, $\rho_{m}$, ϕ is the mode shape of the vibration, *h*, *c*, and *Y* are the membrane density, thickness, viscous solid damping, and Young’s modulus, $\omega_{r}$ is the resonant frequencies of the membrane, and $F_{m}$ is the membrane force.

Assuming quasi-static (considering the large time-scale between vibrations and mixing) conditions, the force on the membrane due to the fluid vibration can be approximated [36,37] by solving the Navier–Stokes equation. The scalar potential flow field, φ, has a solution in the form,

(26) $$\begin{array}{ccc} \varphi & = & \frac{U}{i\omega }\sum_{\phi }K_{\phi }A_{f,\phi }\sinh\;k(\mu ,\omega )(h-z) \end{array}$$

where *k* represents the penetration of the vibration, Ki is the amplitude associated with the mode shape, *h* is the height of the fluid, *z* is the distance from the membrane, μ is the dynamic viscosity, and $A_{f}$ is the geometrical area coefficient of the membrane.

The pressure *P*, therefore, has the following form,

(27) $$\begin{array}{lll} P & = & -\rho_{f}\ddot{\varphi } \end{array}$$

(28) $$\begin{array}{ccc} & = & -j\omega \rho_{f}U\sum_{\phi }K_{\phi }A_{f,\phi }\sinh k(\mu ,\omega )(h-z) \end{array}$$

where $\rho_{f}$ is the density of the fluid.

The force due to the fluid on the membrane, $F_{f}$, can be obtained by integrating the pressure across the membrane area, *A*,

(29) $$\begin{array}{ccc} F_{f} & = & \int_{A}P|z=0dA\\ & = & -j\omega \rho_{f}U\sinh k(\mu ,\omega )h\sum_{\phi }K_{\phi }\int_{A}A_{f,\phi }dA \end{array}$$

(30) $$\begin{array}{ccc} & = & U\rho_{f}\omega h_{f}\left(\omega ,\mu \right)A_{f} \end{array}$$

(31) $$\begin{array}{ccc} h_{f} & = & -j\sinh k(\mu ,\omega )h \end{array}$$

(32) $$\begin{array}{ccc} A_{f} & = & \sum_{\phi }K_{\phi }\int_{A}A_{f,\phi }dA \end{array}$$

where $h_{f}$ is the equivalent height of the fluid on top of the membrane.

The mechanical impedance can be formulated by the summation of the fluid and membrane force (Equations (25) and (29)),

(33) $$\begin{array}{ccc} F_{m}+F_{f} & = & Z_{m}U \end{array}$$

(34) $$\begin{array}{ccc} Z_{m} & = & \omega [\sum_{\phi }\zeta \left(\omega ,\phi ,\rho_{m},h,c,Y\right)\left(\omega_{r}^{2}-\omega^{2}\right)+\rho_{f}h_{f}\left(\omega ,\mu \right)A_{f}] \end{array}$$

Equation (34) shows that mixing degrees, which can be reflected as a change in fluid density $\rho_{f}$ and/or equivalent height $h_{f}$ near the membrane would alter the value of the mechanical impedance. This change would then be reflected in the electrical impedance response, as shown in Kim et al. [17].

### 2.4. Experimental Setup

For the empirical study of the anti-reciprocal property via the gyrator, we designed a simple electro-mechanical system having a permanent magnet, a voice coil, and a loading chamber. The design process was adopted by Kim et al. [17].

Voice coils are thin and the wires lightweight, and a typical application of the voice coil is a dynamic speaker. With the American Wire Gauge (AWG) standards, AWG 30 (cross-section area = 0.0510 (mm ${}^{2}$), resistance = 338 (Ω/km)) or higher is commonly used in the loudspeaker industry. Note that a higher AWG number indicates a thinner wire. A typical material for the voice coil is copper with an insulation coating (i.e., enamel). The main application of the voice coil is a low-power personal gadget (i.e., an earphone); thus, it can be used as a safe and low-cost application. Such a delicate character of the voice coil is suitable for microfluidic applications.

**Figure 2:**
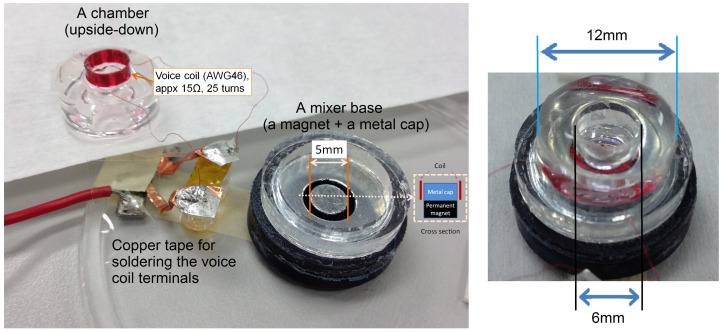
The electromagnetic mixer design. A voice coil is attached to a PDMS-based membrane. A hollow, cylindrical chamber is connected to form a mixer unit. Input signals are driving from the electrical terminals (two ends of the voice coil), and loads vary in mechanical terminals. As shown in the previous study [17], the mechanical loads’ variation, including mixing performance, can be captured at the electrical input terminals.

Once a signal is applied to the coil, the AC magnetic field is generated around the voice coil-loop. Due to the permanent magnet (DC magnetic field), the voice coil moves as it alternates the AC electromagnetic polarities. The coil attached to the polydimethylsiloxane (PDMS) membrane vibrates due to the electromagnetic force at the coil, as depicted in Figure 2. The chamber is used as a fluid container to simulate mechanical loads’ variation.

## 3. Results and Discussion

Based on Ohm’s law, the electrical impedance is defined as voltage over current in Ω or V/A, volts per unit current. It can be interpreted as a one-port network’s transfer function given an output over an input. The voltage is a potential variable in the electrical field; therefore, in electro-mechanical systems, the velocity (flow in the mechanical side) across a gyrator is linked to the voltage term, as shown in Equation (4) (ideal case). Loads in the mechanical front are receptive to the electrical impedance measured; the mechanical loads’ variation should be reflected in the electrical impedance in the electromagnetic systems. The non-ideal case (Equation (13)) contemplates additional AC effects; an EM system is affected by both the induced (AC) and the permanent (DC) magnetic fields. However, when the DC magnetic field governs (i.e., an EM system with a strong magnet and a relatively weak moving coil part), the relationship between the voltage and the velocity shows more similar patterns to each other. In this case, the motional voltage term in Equation (13) dominates the total voltage, Φ, and the electrical impedance response represents the velocity response in a constant current frequency sweep. However, when the transformer impedance becomes more substantial, which is generally the case when we drive the system to its full capability to overcome a sizeable mechanical impedance, we require a constant voltage frequency sweep in order for the velocity response to be represented by the electrical impedance response (see Equation (24)).

**Figure 3:**
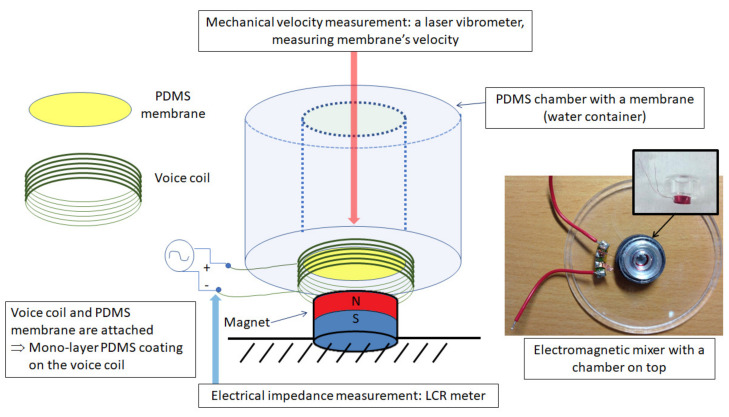
A schematic illustration and a picture to explain the experimental concept. The signal (i.e., sweep frequencies generated by a function generator) is applied to the coil’s terminals. Then, due to the permanent magnet, the electromagnetic force drives the coil to move. An LCR meter is used to take the electrical driving point impedance of the system, and a laser vibrometer light is focused on the membrane to measure the membrane’s velocity.

Taking a case when the permanent DC magnetic field is influential and the transformer impedance is not negligible, we perform both electrical and mechanical experiments and electrical point impedance and mechanical velocity measurements. Figure 3 represents our experimental materials, setting, and methods.

### 3.1. Electrical Impedance Measurement

There are several ways to gauge electrical impedance. One of the methods is to use an LCR meter. For example, the Agilent E4980A Precision LCR Meter was used in this study. Every physically realizable circuit, such as resistors, capacitors, and inductors, has free-loading components. These include undesired resistance in capacitors, capacitance in inductors, and inductance in resistors. Thus, complex impedance representation is required to model a system precisely. The electrical impedance (*Z*) has a real part and an imaginary part. The *Z* can be written in rectangular form as resistance and reactance or in polar style as magnitude and phase. With the LCR meter, one may choose a way to analyse the complex impedance of a physical system.

### 3.2. Mechanical Velocity Measurement

The Polytec CLV3000 3D laser vibrometer (https://www.polytec.com/int/) 3D laser vibrometer has been used for measuring the mechanical velocity of the electromagnetic system. There are four components to drive the laser system: a laser machine, an Data acquisition box (Polytec VIB-E-400 Junction box), a laser controller, and a management computer with the software.

**Figure 4:**
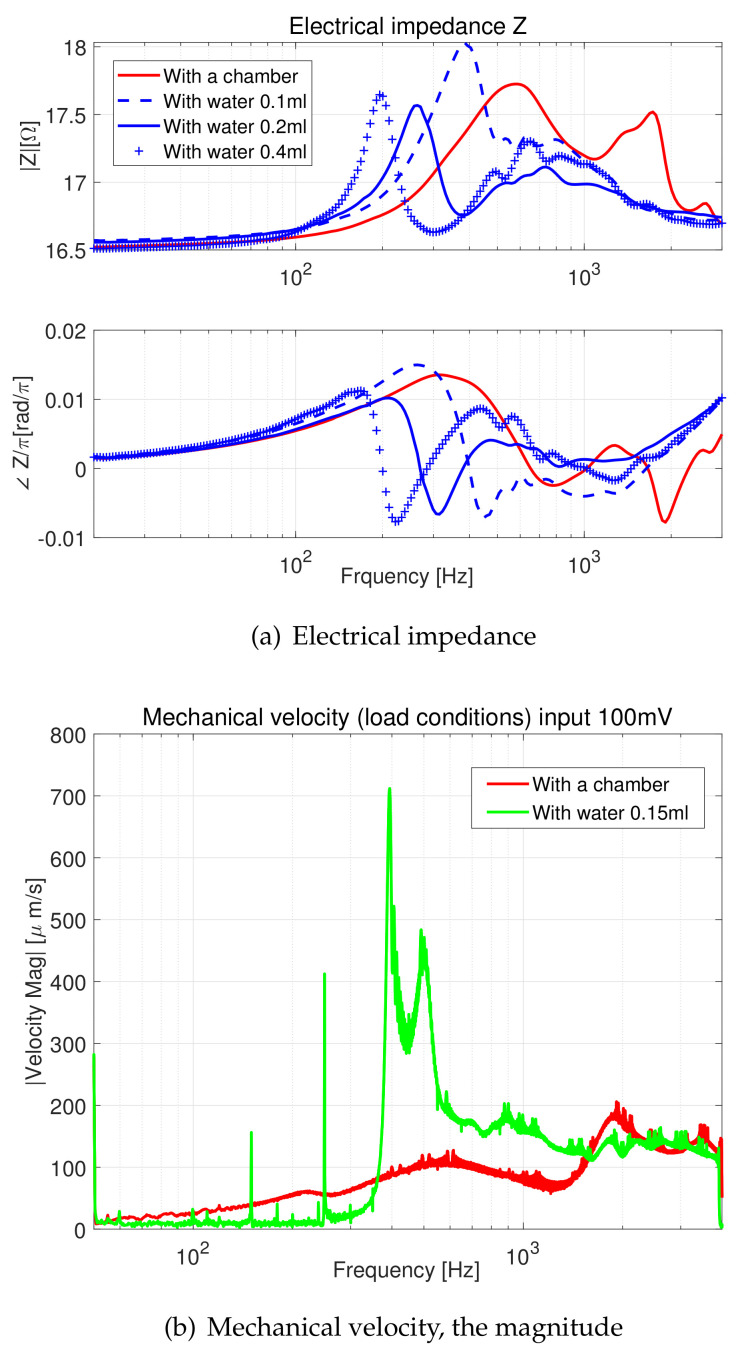
The electrical impedance and mechanical velocity measurement results from the electromagnetic mixer introduced in Figure 2. Two loading conditions are examined: an empty chamber or filled with water. For the electrical impedance measurement, three different water volumes were tested to validate the numeric model suggested in Equations (22) and (23).

Figure 4a,b compares electrical impedance and mechanical velocity data obtained from the same electro-mechanical device introduced in Figure 3. Different amounts of water were loaded into the chamber, then the results were compared from both side experiments.

There were four experimental conditions in the electric impedance data (Figure 4a): a device with a chamber and a device with a chamber loaded with three different amounts of water (change in equivalent fluid height). The same system was tested mechanically. In Figure 4b, the magnitudes of the mechanical velocity (using laser) with two conditions are introduced.

The analogy between the two modalities (electrical and mechanical with the electromagnetic coupling) was discussed in Section 2. Figure 4 carries the empirical evidence; the peak frequency location and overall shape of the first two data of each subfigure corresponded to each other.

A water-filled chamber shifted the damping, as well as the mass of the system. This effect entirely changed the characteristic frequency. The result was reflected well in the electrical impedance data (Figure 4a; the resonance moved to a lower frequency with the mechanical loads’ increment); while in the laser data (Figure 4b), the effect was disturbed due to laser light deflection. Maintaining a clear focus was essential for the laser experiments. However, on account of the electromagnetic system’s dynamic nature, keeping an excellent focal point of the laser light was laborious as the chamber’s membrane was oscillating. Analysing the electrical side data was straightforward by minimizing unnecessary efforts such as filtering out noises and interference. For example, to maximize movement (vibration) with the electromagnetic system, we used 0.15–0.2 mL water loading, and we could drive a 300–400 Hz AC signal, supported by Figure 4a.

As an extension of our previous project, the current study focused on providing theoretical insight into the physical system, our electromagnetic micromixer. Despite the excellent series of results, there are also many exciting challenges for our future work, primarily focusing on mixing technologies, enhancing micromixers’ performance, and design aided by physical simulation. Hosseini Kakavandi et al. [38] investigated mass transfer characteristics in micromixers by varying the junctions and channel shapes of the mixers. Their study demonstrated that the T-shaped mixer’s junction shape and pit diameter critically affected the mass transfer coefficients as chaotic advection was generated by the modification of the mixing channel shape. Chandan Kumar and Nguyen [39] developed a numerical model of magnetic nanoparticles and fluorescent dye under a nonuniform magnetic field. They performed a parametric analysis of the mass transfer process to scrutinize the magnetic field strength and nano-particle size effects in a magnetofluidic micromixer. Their simulation demonstrated that the core stream spread into the upper sheath stream due to magnetoconvection, and their experimental results supported their model simulation. Their work inspired us to investigate our mixer’s mass transfer coefficients based on the current design concept, especially the effect of the voice coil attachment (i.e., position and size concerning the chamber) on the membrane.

## 4. Conclusions

In electromagnetic systems, the magnetic field $\dot{H}$ links the electrical and mechanical sides owing to anti-reciprocal characteristics. The $\dot{H}$ (induced by the conducting current from the coil) is affected by the permanent magnet and changes its polarity (directions). The induced magnetic field and the constant magnetic field define the net force on the coil [34]. Thus, the movement of the coil follows the net force’s direction.

In this study, the electro-mechanic (or electromagnetic) two port system was examined. Starting with electromagnetic theories such as Maxwell’s equation and the Lorentz force law, we investigated a gyrator’s non-ideal formulation. It represented an anti-reciprocal characteristic of the electro-mechanic network. The theory was further explored with the mobility (or dual) analogy” one may choose “the dual analogy with a transformer” or “the impedance analogy with a gyrator” to model an electromagnetic system. An essential benefit of using a gyrator was keeping us from the mystifying dual analogy: it helped an intuitive analysis of the EM network. To our knowledge, this was the first attempt to derive a non-ideal gyrator ever since the gyrator was invented by Tellegen [28]; he suggested the gyrator as the fifth circuit element (along with a resistor, a capacitor, an inductor, and a transformer).

This study expounded on the reason why the electrical impedance data were analogous to the mechanical velocity data. An electromagnetic micromixer was designed and tested to explain the anti-reciprocal nature. Additionally, a benefit from electrical impedance measurement was highlighted. Focusing a laser beam is not easy, especially when the point of focus is the fluid’s surface, which may be sensitive to the surrounding environment such as lights, vibration, noise, etc. These undesignated factors can interfere with the laser light to make it challenging to focus the light on a fixed position. The electrical experiment may be used to overcome this problem, which provided more precise data to characterise the electro-mechanic device.

## Figures and Tables

**Table 1:** Examples of modalities and their conjugate variables. Upper case symbols are used for the frequency domain variables except in the electromagnetic (EM) case, as its traditional notation uses capital letters for the time-domain analysis (i.e., E(t)=−∇ϕ(t), where ϕ(t) is the electric scaler potential and the voltage in the time domain.

Modality	Conjugate Variables (Vector in Bold)	
	Generalised Force (Unit)	Flow (Unit)
Electric	Voltage (Φ) (V)	Current (*I*) (A)
Mechanic	Force (F) (N)	Particle velocity (U) (m/s)
Acoustic	Pressure (P) (N/$m^{2}$)	Volume velocity (V) (1/ms)
Electromagnetic	Electric field (E) (V/m)	Magnetic field (H) (A/m)
